# Sensitive and Selective Detection of Clenbuterol in Meat Samples by a Graphene Quantum Dot Fluorescent Probe Based on Cationic-Etherified Starch

**DOI:** 10.3390/nano12040691

**Published:** 2022-02-19

**Authors:** Huanyu Xie, Cairou Chen, Jiansen Lie, Ruiyun You, Wei Qian, Shan Lin, Yudong Lu

**Affiliations:** 1 Fujian Provincial Key Laboratory of Advanced Oriented Chemical Engineer, Fujian Key Laboratory of Polymer Materials, College of Chemistry and Materials Science, Fujian Normal University, Fuzhou 350007, China; zou82465zou@163.com (H.X.); m17875511657@163.com (C.C.); 17875785934@163.com (J.L.); 2 Research Centre of Wetlands in Subtropical Region, School of Geographical Sciences, Fujian Normal University, Fuzhou 350007, China; fjnuw@fjnu.edu.cn; 3 Jiangxi Province Key Laboratory of Polymer Micro/Nano Manufacturing and Devices, East China University of Technology, Nanchang 330013, China; linshanls2019@163.com

**Keywords:** nitrogen-doped graphene quantum dots, fluorescence, cationic-etherified starch, fluorescence quenching, clenbuterol

## Abstract

The use of clenbuterol (CLB) in large quantities in feedstuffs worldwide is illegal and potentially dangerous for human health. In this study, we directly prepared nitrogen-doped graphene quantum dots (N-GQDs) by a one-step method using cationic-etherified starch as raw material without pollution, which has the advantages of simple, green, and rapid synthesis of N-GQDs and high doping efficiency of nitrogen elements, compared with the traditional nitrogen doping method of reacting nitrogen source raw material with quantum dots. The N-GQDs synthesized by cationic etherification starch with different substitution degrees (DSs) exhibit good blue-green photoluminescence, good fluorescence stability, and water solubility. By comparing the fluorescence emission intensity of the two methods, the N-GQDs prepared by this method have higher fluorescence emission intensity and good fluorescence stability. Based on the static quenching mechanism between CLB and N-GQDs, a fluorescent probe was designed to detect CLB, which exhibited a wide linear range in the concentration range of 5 × 10^−10^~5 × 10^−7^ M (R^2^ = 0.9879) with a limit of detection (LOD) of 2.083 × 10^−13^ M. More excitingly, the N-GQDs fluorescent probe exhibited a satisfactory high selectivity. Meanwhile, it can be used for the detection of CLB in chicken and beef, and good recoveries were obtained. In summary, the strategic approach in this paper has potential applications in the detection of risky substances in the field of food safety.

## 1. Introduction

Clenbuterol (CLB) is a substance in the class of β_2_ adrenergic receptor agonists. It was often used as a bronchodilator for the clinical treatment of croup, and it can act in the vascular system of animals, affecting their metabolism and thus affecting their normal development and lean muscle mass [1,2]. CLB residues in meat products can enter the human body through the food chain can cause poisoning phenomena such as headache, dizziness, chest tightness, palpitations, skeletal muscle tremors, numbness of the limbs, etc., which can lead to life-threatening in severe cases [3,4]. The addition of CLB in livestock has been strictly restricted, but there are still some countries in Southeast Asia, where CLBs are used illegally to promote the rapid growth of meat animals. Therefore, there is an urgent need to establish an accurate, sensitive, and selective method for the detection of clenbuterol residues in meat foods.

To date, several analytical methods have been established, such as liquid chromatography–mass spectrometry (LC–MS) [5], enzyme-linked immunosorbent assay (ELISA) [6,7], gas chromatography coupled with mass spectrometry (GC–MS) [8], surface-enhanced Raman scattering (SERS) [9,10], and electrochemical detection [11]. However, these methods require expensive instruments and complex pretreatment processes, which hinder the widespread application. ELISA methods, although easy to operate and highly sensitive, require considerable work due to their dependence on the mutual specificity of antigen–antibody interaction. Electrochemical methods have the disadvantage of relatively poor stability. Although the SERS method does not require a complicated pretreatment process and short determination time, it has the disadvantages of being unable to detect quantitatively and having poor stability. The LC–MS method is the most common due to its short chromatographic run time. However, the complex pretreatment method may cause, organic contamination, leading to a large error in the accuracy of detection. In contrast, fluorescence spectroscopy is a method with the advantages of very low cost, simple operation, high sensitivity, selectivity, and ease of detection.

Graphene quantum dots (GQDs) are among hot topics of research in recent years and have the advantages of metal quantum dots in terms of electrical conductivity, thermal conductivity, and low toxicity, compared with metal quantum dots [12]. Currently, there are two methods for the preparation of GQDs—namely, the top-down method [13,14] and the bottom-up method [15,16]. Top-down methods include electrochemical stripping [17], oxidation [18], microwave radiation [19], etc. These methods require expensive equipment and high costs. Bottom-up methods include hydrothermal [20] and chemical synthesis [21], which have the advantages of simple synthesis and low cost; however, the reaction process usually involves acids and bases, which can lead to disadvantages such as complicated post-treatment. The use of biomass materials for the synthesis of GQDs is a recent hot area of research, with scientists using biomass materials such as green tea [22], carrots [23], graphite waste [22], orange juice [24], honey [25], rice [26] to prepare GQDs, N-GQDs, and other element-doped GQDs.Cationic starch is a starch derivative with a positively charged surface that has high dispersibility, good water solubility, degradability, and sterilization and is used in a wide range of applications. Previously, the preparation of quantum dots from cationic starch has been less frequently reported, as cationic starch has more amino groups on its surface, and the synthesized quantum dots have better fluorescence and stability than those synthesized directly from starch. Traditional methods for the preparation of nitrogen-doped quantum dots are mainly through the physical mixing of nitrogen sources and carbon substrates, followed by the chemical synthesis of N-GQDs. The method in this paper uses cationic-etherified starch as the raw material for the direct one-step synthesis of N-GQDs, which reduces the loss of nitrogen during the preparation of N-GQDs because the cationic-etherified starch contains more amino groups, and the amino groups are linked to the starch molecules by chemical grafting. The doping of elements such as nitrogen, sulfur, and chlorine has modifying effects on the properties of GQDs, and N-GQDs increase the positive charge density of the C atoms in graphene due to the five valence electrons and the N atoms. N atomic chemical dopants can provide more active centers for GQDs, allowing them to exhibit a high degree of fluorescence emission and good stability properties [27,28].

**Scheme 1:**
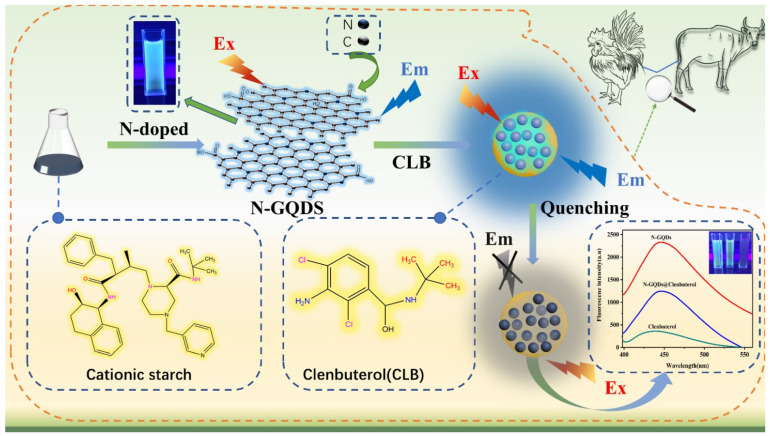
Schematic diagram of the detection of clenbuterol by N-GQD fluorescent sensors and its application to real samples.

Here, we propose a fluorescent probe for the determination of CLB by using cationic starch as the raw material for the preparation of N-GQDs, which have better fluorescence effects than those synthesized directly from starch, and the presence of CLB will quench the N-GQDs. The N-GQDs fluorescent probes prepared in this paper provide an efficient, rapid, and sensitive method for the detection of clenbuterol and have been successfully applied to the detection of CLB in chicken and beef (Scheme 1). To our knowledge, few studies have sought to prepare N-GQDs using cationic-etherified starches and use them for the detection of CLB and their application to the analysis of CLB in real samples. This strategy holds good promise for use in the detection of hazards in the field of food safety.

## 2. Experimental Section

### 2.1. Chemicals and Materials

Cationic starch with different degrees of substitution and Tapioca starch was purchased from Aladdin Biochemistry (Shanghai, China). Clenbuterol was purchased from Shanghai Aladdin Biochemical Co. Ltd., and beef and chicken (within shelf life) were purchased from Metro supermarkets (Fuzhou, China). Glycine (Gly), cysteine (Cys), tyrosine (Tyr), arginine (Arg), lysine (Lys), and glutamic acid (Glu) were purchased from Aladdin Chemical Reagents (Shanghai, China). Clenbuterol, terbutaline, ractopamine, cimatro, and salbutamol were purchased from Shanghai Maclean’s Biochemical Technology Co Ltd. (Shanghai, China). NaCl, KCl, CaCl_2_, MgCl_2_ were purchased from Aladdin Chemical Reagents (Shanghai, China). Analytical grade chemicals and reagents were used in the experiment, and the solutions were prepared with distilled water (Guozhiyuan Y1/2-10UV, Changsha, China).

### 2.2. Instruments

UV–vis absorption spectra were obtained using a UV-1800 UV-vis spectrophotometer (Shimadzu, Kyoto, Japan). X-ray photoelectron spectroscopy (XPS) images were recorded using a Thermo Scientific K-alpha X-ray photoelectron spectrometer (Shimadzu, Manchester, UK). Transmission electron microscopy (TEM) images were obtained using a Tecnai-G20 (FEI, Hillsboro, OR, USA). Particle size distributions and zeta potentials were obtained using a model ZS90 Zeta-sizer nanodevice (Malvern Panalytical, Malvern, UK). Fluorescence spectra were obtained using a Hitachi F-7000 fluorescence spectrophotometer (Hitachi, Tokyo, Japan). pH tests were obtained using a PHC-3C pH meter (YOKE, Shanghai, China). Fluorescence lifetime tests were obtained using an FLS1000 Edinburgh steady-state/transient fluorescence spectrometer (Edinburgh Inc., Livingston, UK). Electron images under N-GQDs UV lamp irradiation were obtained using a ZF-5 UV lamp (Mingren Electronic Instruments, Wenzhou, China).

### 2.3. Preparation of N-GQDs and GQDs

Cationic etherified starch with degrees of substitution (DS) of 0.2, 0.1, 0.05 were purchased as carbon sources to prepare cationic-etherified starch-based N-GQDs as follows: First, 0.6 g of cationic-etherified starch was weighed on a balance, put into a flask, added to a magnetic rotor, heated to 60 °C, and stirred for 20 min to dissolve it. After dissolution, the above solution was transferred to a Teflon autoclave and put into a high-temperature oven for 6 h (the high-temperature oven was heated to 160 °C in advance); after the reaction was finished, it was cooled naturally to room temperature, transferred to a centrifuge tube, and centrifuged at 12,000 r/min for 30 min; then, the precipitate was removed, the solution was transferred to a new centrifuge tube, and the N-GQDs were filtered through a 220 nm filter membrane. After filtering, the light brown liquid was transferred to a dialysis bag and dialyzed for 48 h using a 1000 DA dialysis bag, and the deionized water was changed once every 6 h. The light-brown liquid obtained after the completion of dialysis was the N-GQDs product, which was stored in a refrigerator at 4 °C for the next tests.

The same experimental conditions and steps as above were used to prepare GQDs from tapioca starch, to obtain an aqueous solution of GQDs, which was stored in a refrigerator at 4 °C for the next experiment; N-GQDs were prepared using tapioca starch and urea as the carbon and nitrogen sources, respectively, as follows: First, 0.6 g of tapioca starch and 0.36 g of urea were added to 25 mL of deionized water and heated to 60 °C. After stirring for 20 min, the mixture was transferred to a polytetrafluoroethylene reactor for hydrothermal reaction. The experimental conditions and experimental steps were the same as those described above to obtain N-GQDs solutions based on tapioca starch and urea, which were placed in a refrigerator at 4 °C for the next experiments.

### 2.4. Calculation of Fluorescence Lifetime

In this study, the Edinburgh FSL1000 lifetime and steady-state spectrometer were used to determine the fluorescence lifetime of the product. The lifetime decay curve is calculated according to the following double exponential function Y_(t)_ (Equation (1)), and the average fluorescence lifetime τ_3_ of the test substance is estimated according to Equation (2):

Y_(t__)_ = A_1_ exp(−t/τ_1_) + A_2_ exp(−t/τ_2_) (1)

τ_3_ = (A_1_ τ_1_^2^ + A_2_ τ_2_^2^)/(A_1_ τ_1_ + A_2_ τ_2_) (2)

where τ_1_ and τ_2_ are the fitted lifetimes of the test results; A_1_ and A_2_ denote the fractional contributions of the time-resolved fluorescence decay lifetimes τ_1_ and τ_2_, respectively, while τ_3_ denotes the average fluorescence lifetime of the test substance.

### 2.5. Detection of CLB

A series of CLB standard solutions of different concentrations were placed in a centrifuge tube; then, 30 μL of CLB standard solutions of different concentrations, 3 mL of already diluted N-GQDs solution, and pH 7.0 dilution buffer were added, diluted to 5.0 mL, and shaken well; the reaction was carried out at room temperature for 5 min. The fluorescence emission was recorded at an excitation wavelength of 360 nm and a slit of 10 nm on an F-7000 fluorescence spectrophotometer. The fluorescence intensity (F) of the solution after the addition of CLB and the fluorescence intensity (F_0_) of the N-GQDs solution before the addition of CLB were measured, and the error curves were plotted.

### 2.6. Selective Assessment of CLB by Fluorescent Probes of N-GQDs

Selectivity in fluorometric assays is critical. To further demonstrate the selectivity of the N-GQDs fluorescence system for CLB, we used the same method to measure other structurally similar substances including terbutaline, ractopamine, cimatro, salbutamol, and possibly amino acids such as glycine (Gly), cysteine (Cys), tyrosine (Tyr), arginine (Arg), lysine (Lys), glutamic acid (Glu), and Na^+^, K^+^, and Mg^2+^ (10 times the concentration of CLB). The relative fluorescence emission intensity (F/F_0_) of the fluorescent system was measured after the addition of the above substances, respectively.

### 2.7. Determination of CLB in Real Samples

To validate the feasibility of the method for the detection of CLB in real food samples, fresh beef and chicken samples were pretreated according to the national standard GB/T 6682-2008 as follows: First, 5.0 g samples of fresh beef and chicken were chopped and blended for 2 min using a blender; then, the meat was placed in a centrifuge tube with 8 mL of sodium acetate buffer solution and aromatic sulfate lyase, hydrolyzed in an oven at 37 °C for 12 h, then the above mixture was put into a high-speed centrifuge at 9000 r/min. After centrifugation, the supernatant was added to 0.1 M perchloric acid, and the pH was adjusted to 11 with sodium hydroxide. In total, 10 mL of saturated sodium chloride solution, and 10 mL of isopropyl acetate were added to the above solution. Finally, the clean supernatant was evaporated by nitrogen blowing, and the samples were centrifuged at 5000 rpm for 15 min to obtain the pretreated chicken and beef samples. After completion of the pretreatment, spiked recovery tests were carried out on beef and chicken samples. Different concentrations (2.5 × 10^−6^, 1 × 10^−6^, 2.5 × 10^−7^ M) of 50 μL of CLB solution were added to the appealed pretreated chicken solution and beef solution, followed by 3 mL of N-GQDs (10^−5^ M) for 10 min at room temperature, perform three replicate experimental tests. The N-GQDs@CLB fluorescent system is transparent and noncloudy after the addition of CLB. Finally, fluorescence emission intensity experiments were performed on the obtained fluorescent system solutions using a fluorescence spectrophotometer.

## 3. Results and Discussion

### 3.1. Characterization of N-GQDs

**Figure 1:**
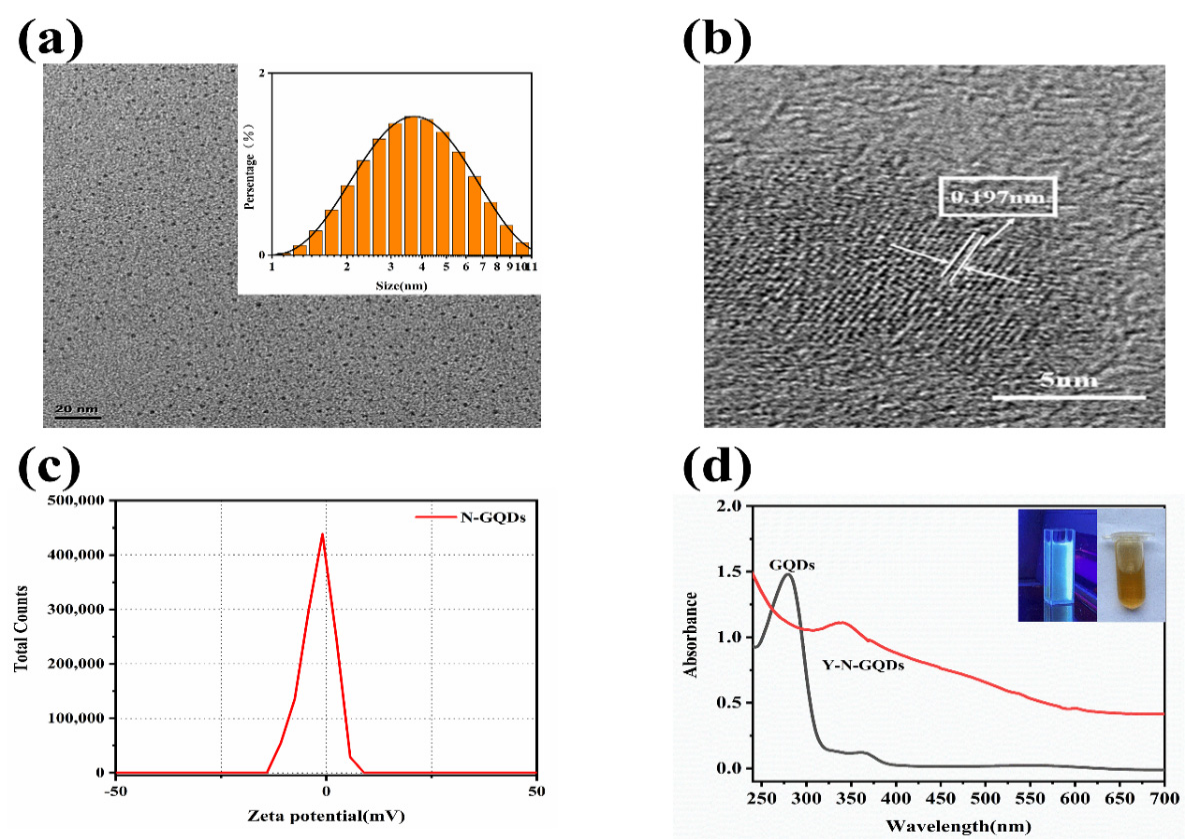
(**a**) TEM image of N-GQDs and the inset of 1a is size distribution; (**b**) HRTEM image of N-GQDs; (**c**) potential distribution of N-GQDs, N-GQDs with a degree of substitution of 0.2; (**d**) UV–vis absorption spectrum of N-GQDs and GQDs. The inset of 1d shows an aqueous solution of N-GQDs in sunlight (**right**) and 365 nm UV light (**left**).

Transmission electron microscopy (TEM) images of N-GQDs (DS = 0.2) are shown in Figure 1a. The results show that the N-GQDs with DS of 0.2 were well dispersed and spherical; from the inset of Figure 1a, it can be seen that the size distribution of N-GQDs is 3–4 nm, with an average particle size of 3.6 nm. The high-resolution transmission electron microscopy (HRTEM) images of N-GQDs reveal a clear lattice structure of the material, and Figure 1b reveals a clear carbon lattice stripe, with a lattice spacing of 0.197 nm, which corresponds to the (100) eigenfaces of graphitic carbon being matched [29]. This confirms that the N-GQD was narrowly distributed, uniformly dispersed, and a well-formed crystalline. We also tested the zeta potential of the N-GQDs, as shown in Figure 1c, and the prepared N-GQDs were weakly negative and close to electrically neutral.

We also studied the UV absorption spectra of N-GQDs and GQDs. The peaks appearing at 272 and 367 nm in the UV patterns of GQDs correspond to π–π* and n–π* jumps of aromatic C=C, respectively [30,31]. According to Figure 1d, GQDs have absorption peak signals at 367 nm, while N-GQDs show absorption peak signals at 340 nm, which are blue-shifted by 27 nm in comparison with the absorption peaks of GQDs; this provides an important basis for the successful synthesis of N-GQDs. The inset of Figure 1d shows that the N-GQD solution appears yellowish brown under sunlight and blue under irradiation using a 365 nm UV lamp.

**Figure 2:**
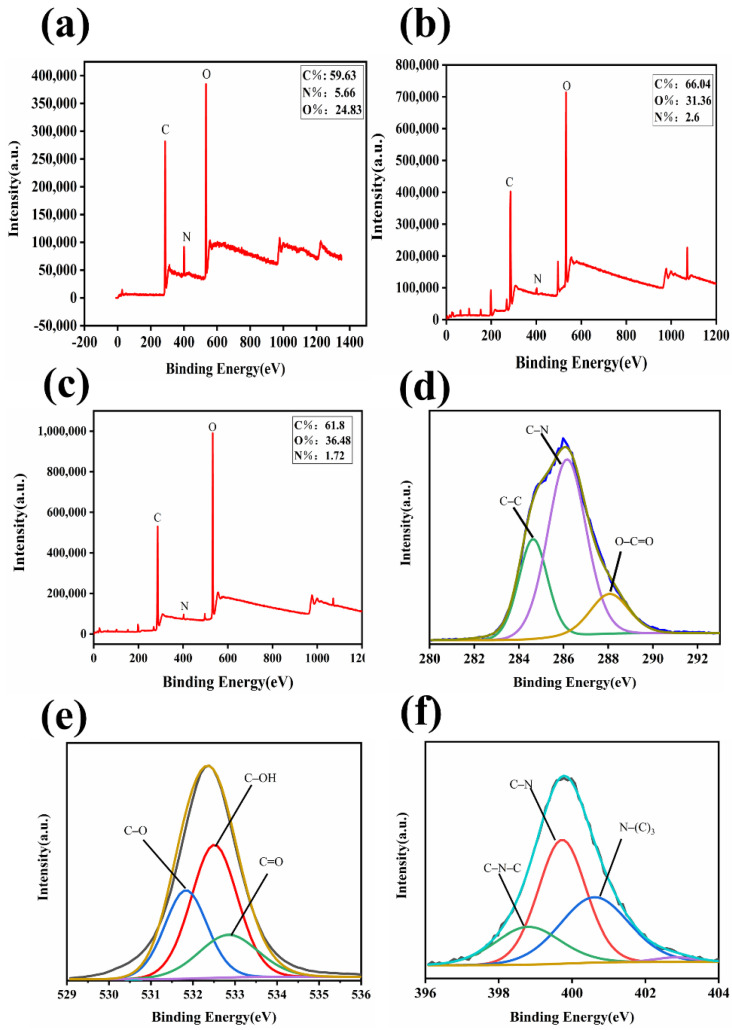
(**a**) Full-scan range of XPS spectra of synthetic N-GQDs (DS: 0.2); (**b**) full-scan range of XPS spectra of synthetic N-GQDs (DS: 0.1); (**c**) full-scan range of XPS spectra of synthetic N-GQDs (DS: 0.05); (**d**) fluorescence stability of N-GQDs. X-ray photoelectron spectroscopy (XPS) spectrum of C 1 s (**d**), O 1 s (**e**), and N 1 s (**f**), respectively.

Furthermore, as X-ray photoelectron spectroscopy (XPS) is commonly used to determine the elemental composition and structural characteristics of N-GQDs, we performed XPS tests on N-GQDs prepared from different DSs (0.2 (Figure 2a), 0.1 (Figure 2b), 0.05 (Figure 2c)) to explore the effect of DS on the elemental N content of the synthesized N-GQDs; as can be seen from the comparison of images in Figure 2a–c, the higher the DS degree, the higher the N content of the synthesized N-GQDs. Therefore, we chose N-GQDs prepared with a DS of 0.2 as the raw material to be used as the fluorescent probes in this study.

As shown in Figure 2a, the XPS measured spectra of N-GQDs (DS = 0.2) shows C1 s, O1 s, and N1 s signals with 59.43%, 5.66%, and 24.83% percentages of C, N, and O elements, which demonstrates the successful doping of N elements onto GQSs during the synthesis process. High-resolution C1s spectra of N-GQDs in Figure 2d show three signal peaks for the C=C (284.8 eV), C–N (286.2 eV), and O–C=O (288.2 eV) functional groups, according to the reported articles, confirming their presence [32]. Figure 2e shows the O1s spectra of N-GQDs, with C–O (531.8 eV), C–OH (532.6 eV), and O=C (532.9 eV) [33]; Figure 2f shows high-resolution XPS spectra of N1s, indicating the presence of N–C (399.8 eV), N–(C)_3_ (400.9 eV), and C–N–C (398.8 eV) on the surface of N-GQDs [34]. Therefore, it can be concluded that N elements were successfully doped into GQDs, resulting in N-GQD fluorescent probes.

### 3.2. Fluorescence Spectroscopy of N-GQDs

**Figure 3:**
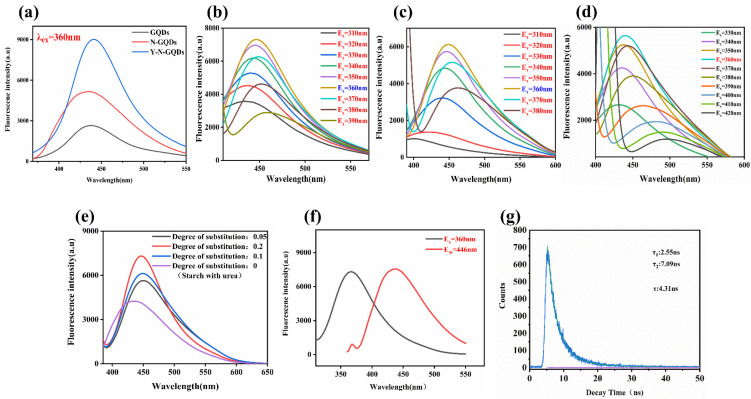
(**a**) Fluorescence emission intensity of N-GQDs or GQDs synthesized from different raw materials at an excitation wavelength of 360 nm; (**b**–**d**) emission spectra of N-GQDs prepared from cationic starch with different degrees of substitution (0.2, 0.1, 0.05) at different excitation wavelengths between 310 and 390 nm, respectively (in steps of 10 nm); (**e**) emission fluorescence intensity of N-GQDs prepared from cationic starches with different degrees of substitution at an excitation wavelength of 360 nm; (**f**) PL excitation and emission spectra of the N-GQDs; (**g**) N-GQD fluorescence decay profiles.

In order to prepare N-GQDs with excellent fluorescence, we prepared N-GQDs from raw materials with different degrees of substitution (DS) (0.2, 0.1, 0.05) and then tested the fluorescence properties. As shown in Figure 3a, we compared the fluorescence intensity of graphene quantum dots prepared from three raw materials (tapioca starch (GQDs), cassava starch and urea (N-GQDs), and cationic-etherified starch (Y-N-GQDs)), at the same excitation wavelength of 360 nm, and found that N-GQDs prepared from cationic-etherified starch had higher fluorescence intensity. Figure 3b–d show N-GQDs prepared from cationic-etherified starch with DS of 0.2 (b), 0.1 (c), and 0.05 (d), respectively, which were subjected to fluorescence spectroscopy, and the maximum fluorescence emission intensity appeared at 446 nm, at an excitation wavelength of 360 nm, showing a good fluorescence effect. N-GQDs prepared from cationic-etherified starches with different degrees of substitution had fluorescence properties that were wavelength-dependent, independent of the degree of substitution of the cationic starch.

As shown in Figure 3e, the fluorescence intensities of N-GQDs prepared from different substitution degree (DS) raw materials were compared at the emission wavelength of 446 nm at an excitation wavelength of 360 nm. The results show that the fluorescence emission intensity of N-GQDs prepared from the more substituted cationic-etherified starches at the same excitation wavelength and preparation conditions is higher. Therefore, we chose DS (0.2) as the raw material for the N-GQDs as the fluorescent probe for this experiment. In Figure 3f, we can see that the excitation wavelength of N-GQDs is 360 nm, and the emission wavelength is 446 nm. Additionally, as shown in Figure 3b, the fluorescence emission wavelength of N-GQDs has a wavelength red-shift variation in the excitation wavelength and intensity range of 310–390 nm, and the fluorescence emission intensity reaches the maximum at 446 nm upon 360 nm excitation. We measured the fluorescence decay curves of the N-GQDs using an Edinburgh FLS1000 lifetime and steady-state spectrometer. As shown in Figure 3g, we performed a second-order fluorescence lifetime test with fluorescence lifetimes of N-GQDs τ_1_ = 2.55 ns and τ_2_ = 7.09 ns; the fluorescence lifetime (τ_3_) of the synthesized N-GQDs was calculated to be 4.31 ns on average. Therefore, it can be concluded from the above characterization that N-GQDs have excitation wavelength dependence and good photoluminescence properties.

### 3.3. Effect of pH and Temperature on the Fluorescence Intensity of N-GQDs

**Figure 4:**
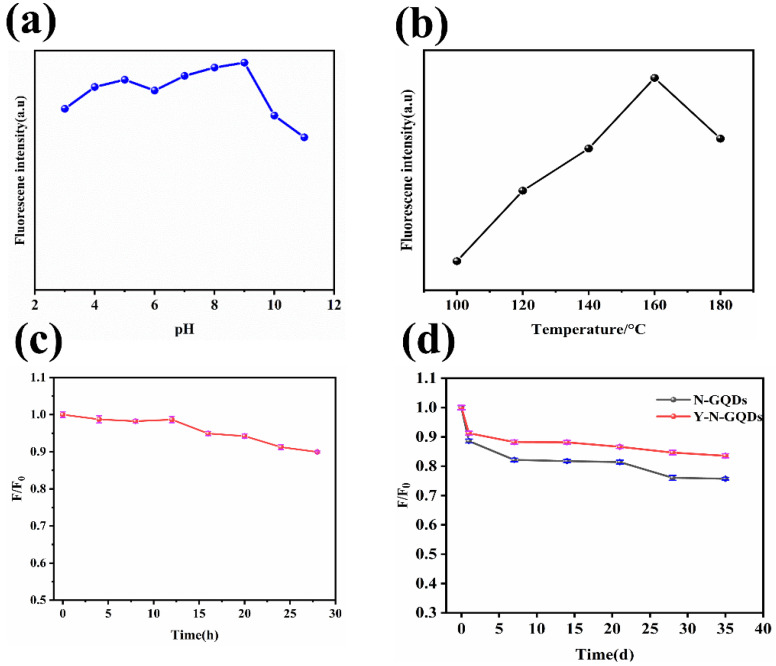
(**a**) Effect of different pH conditions on the fluorescence emission intensity of N-GQDs; (**b**) effect of different reaction temperatures on the fluorescence emission intensity of N-GQDs; (**c**,**d**) comparison of the fluorescence stability of Y-N-GQDs prepared from cationic-etherified starch with N-GQDs prepared from tapioca starch and urea.

We investigated the effect of experimental conditions (pH, temperature) on the fluorescence intensity of N-GQDs (DS = 0.2). The effect of pH on fluorescence properties of N-GQDs was analyzed, the results of which are shown in Figure 4a. With fixed parameters in terms of other reaction conditions, the effect of pH on the fluorescence emission intensity of N-GQDs was studied. Figure 4a shows that the fluorescence intensity starts to increase in the pH range of 2–5 and starts a decrease when the pH is in the range of 5–6; when the pH is in the range of 6–9, the fluorescence intensity starts to increase again and remains relatively stable; when the pH is greater than 9, the fluorescence intensity starts to show a substantial decrease. The results of this study show that the fluorescence intensity of strong acids and alkalis is not significantly reduced. In addition, strong acids and bases reduce the fluorescence behavior of N-GQDs. Due to the presence of a large number of groups on the surface of N-GQDs, the presence of many charge ions in strongly acidic and alkaline environments can lead to the occurrence of surface effects that affect the radiation leap efficiency of N-GQDs, and the corresponding changes in the fluorescence emission intensity of N-GQDs occur. Therefore, we chose a neutral pH state as the parameter for the next experiments.

Figure 4b plots the fluorescence emission curves of the N-GQDs prepared at different reaction temperatures. As shown in Figure 4b, the fluorescence intensity increases with the increase in reaction temperature, and the fluorescence intensity reaches a peak at 160 °C. When the reaction temperature is higher than 160 °C, fluorescence intensity starts to decrease. This mechanism is possible due to the dehydration reaction of starch to form glucose, while oligosaccharides are dehydrated at higher temperatures and undergo intermolecular cross-linking. Under hydrothermal conditions, the formyl group undergoes a dehydration reaction with the hydrocarbon group. Finally, cyclic condensation produces GQDs [35]. Therefore, the best fluorescence of N-GQDs was prepared at a reaction temperature of 160 degrees C and an ambient pH = 9.

We also investigated the stability of the synthesized N-GQDs, as shown in Figure 4c. The fluorescence intensity of N-GQDs remains basically unchanged for 28 h, and the decrease in fluorescence intensity at 28 h is only less than 10%. As shown in Figure 4d, the N-GQDs prepared by cationic-etherified starch (Y-N-GQDs) have better fluorescence stability than the urea-doped N-GQDs, and the fluorescence intensity of Y-N-GQDs decreases only 11% in 35 days, compared with 0 days, while the fluorescence intensity of N-GQDs decreases 22%, compared with 0 days. Therefore, the N-GQDs prepared in this study have good fluorescence stability.

### 3.4. Fluorescence Response of N-GQDs to CLB

**Figure 5:**
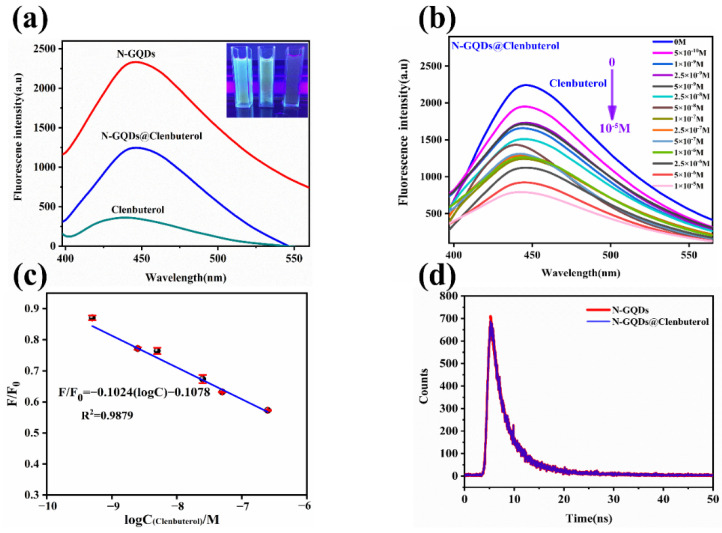
(**a**) Fluorescence emission spectra of 0.2 mg mL^−1^ N-GQDs in solution with and without the addition of 10^−5^ M CLB. (**b**) Fluorescence emission spectra of different concentrations of CLB (0–10^−5^ M) when added to N-GQDs. (**c**) Linear plot of the degree of variation of the system fluorescence intensity, F/F_0_, versus log C(CLB) concentration values (CLB concentrations of 5 × 10^−10^ to 5 × 10^−7^ M), with F and F_0_ being the fluorescence emission intensity in the presence or absence of CLB in the N-GQDs solution, respectively. (**d**) Fluorescence emission decay curve of 0.2 mg ml^−1^ N-GQDs and N-GQDs@CLB (10^−5^ M).

We investigated the effect of the fluorescence response of the N-GQDs fluorescent probe to CLB. Figure 5a shows the fluorescence emission spectra of N-GQDs and N-GQDs and CLB solutions at the same excitation wavelength of 360 nm, and it can be seen that direct fluorescence spectroscopy of the CLB solution is essentially devoid of fluorescence emission intensity. A significant quenching of the fluorescence emission intensity of the N-GQDs fluorescence system occurs after the addition of 50 μL CLB (10^−5^ M, with a quenching efficiency of about 49%, and CLB could significantly quench the fluorescence intensity of the N-GQD solution. Different concentrations of CLB were added to the N-GQDs probe to test its performance in detecting CLB. Figure 5b shows that, with the increase in CLB concentration, the fluorescence intensity of N-GQDs emission shows a decreasing signal and a fluorescence quenching effect, indicating that CLB can effectively quench the fluorescence intensity of N-GQDs. The linearity of the correlation between the fluorescence intensity of the fluorescent sensor and the negative logarithm of CLB can be seen in Figure 5c. There is a good linear relationship (R^2^ = 0.9879) between the relative fluorescence intensity of the system (F/F_0_), and the concentration over the range of CLB concentrations (5.0 × 10^−10^~5.0 × 10^−7^ M). The linear regression equation is F/F_0_ = −0.1024(log C) − 0.1078, where C is the CLB concentration. The limit of detection (LOD) is 2.083 × 10^−13^ M according to the 3 σ/s rule (where s is the slope of the linear calibration plot, and σ is the sum of the standard deviations of the blank signals). This N-GQDs fluorescent probe assay has a wide linear range and a low detection limit. Thus, the present study provides an achievable fluorescence method for the rapid and semi-quantitative detection of CLB.

### 3.5. Assay Selectivity

**Figure 6:**
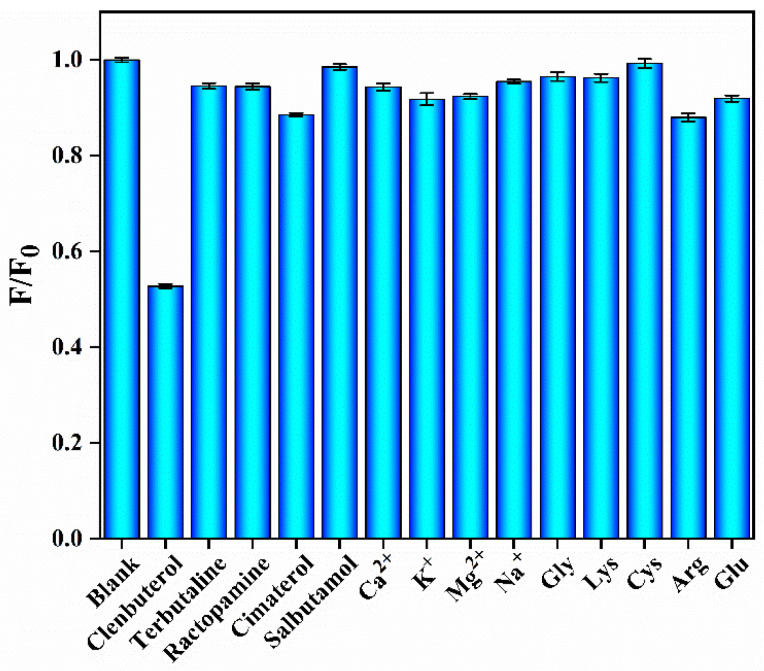
Testing the selectivity of N-GQDs for CLB in the coexistence of different interfering substances (10^−5^ M for the other analytes and 10 times the concentration of CLB for the metal ions).

To investigate the selectivity of the N-GQDs@CLB assay system by testing different potential interfering substances (terbutaline, ractopamine, cimatro, salbutamol (Sal)), amino acids (cysteine (Cys), lysine (Lys), glutamic acid (Glu), glycine (Gly), arginine (Arg)) and metal ions (Ca^2+^, Mg^2+^, Na^+^) were subjected to interference experiments under coexistence conditions, and the selectivity of N-GQDs for CLB was investigated by measuring the relative fluorescence intensity (F/F_0_) of N-GQDs solutions with 50 μL (10^−5^ M) of different substances. As shown in Figure 6, the fluorescence intensity of the system changes negligibly under the conditions of coexistence of the fluorescent probe with other interfering substances. In contrast, CLB has a significant fluorescence quenching effect on the fluorescent system of N-GQDs. It can be concluded that N-GQDs have good selectivity for CLB.

### 3.6. Detection for CLB Content in Real Samples

To assess the feasibility of fluorescent probes for the detection of CLB in real food samples by N-GQDs, CLB was detected in beef and chicken samples using the N-GQDs@CLB probe. Recovery experiments were carried out on CLB in real samples using the standard addition method. As shown in Table 1, the spiked recoveries in chicken meat samples range from 98.4% to 105.2%, with relative standard deviations (RSDs) of 8.26–10.32%. The recoveries in the beef samples range from 99.2% to 108.8% with relative standard deviations (RSDs) of 6.90–10.25%. The above results show that the proposed strategy is feasible and applicable and that the fluorescent probe provides a viable method for the sensitive, rapid, and highly sensitive detection of CLB in real food samples.

In addition, we further compared the method of this paper with other reported methods for CLB detection (Table 2), and it can be clearly concluded that the N-GQD fluorescent probe of this research is a new method with higher sensitivity and can detect CLB quickly and easily.

### 3.7. Potential Quenching Mechanism Study

With the addition of CLB to the fluorescent system of N-GQDs, the fluorescence intensity of the system decreased. This indicates that there is a quenching mechanism between CLB and N-GQDs. We investigated the potential quenching mechanism. XPS studies showed that there are many hydrophilic groups (–COOH, –OH, –NH_2_) on the surface of the synthesized N-GQDs, and some hydrocarbon and amino groups are present in the molecular structure of CLB, the oxygen atoms on the surface of the carboxyl and amino groups are strongly electronegative, and the formation of intermolecular hydrogen bonds is possible. Once hydrogen bonds are formed, N-GQDs have high sensitivity and selectivity for CLB and may lead to a reduction in the fluorescence intensity of N-GQDs; this mechanism is similar to that reported by Zhang et al. [42]. Furthermore, the above intermolecular hydrogen bond formation is not sufficient to explain the fluorescence quenching of N-GQDs due to CLB. Liu et al. found that the fluorescence of GQDs could be quenched by CLB through the diazotization-coupling reaction. The decrease in fluorescence intensity was explained by the change in the absorption spectrum of GQDs after the addition of CLB to the diazotization reaction, and the quenching mechanism was investigated by the change in fluorescence lifetime before and after the addition of CLB [43]. We measured the fluorescence lifetime of the N-GQDs solution before and after the addition of CLB (50 μL, 10^−5^ M), and the test instrument was Edinburgh FLS 1000, as seen in Figure 4d. The fluorescence lifetime of the N-GQDs fluorescence system with the addition of CLB increased from 4.31 ns to 4.58 ns, which shows that the fluorescence of CLB quenched N-GQDs is based on the static mechanism of burst extinction.

## 4. Conclusions

In summary, we used cationic-etherified starch to synthesize N-GQDs in one step and developed a direct, simple, highly sensitive, and selective fluorescent probe for the detection of CLB and demonstrated the application of this fluorescent probe for the detection of potential CLB in food samples. The advantage of using cationic-etherified starch for the direct synthesis of N-GQDs is that the conventional method of nitrogen doping does not require the use of other carbon sources and carbon substrates; due to a large number of amino groups within the cationic-etherified starch, the amino groups are directly linked to the starch molecules through chemical bonding, allowing the direct synthesis of N-GQDs with good nitrogen doping. This method can reduce the loss of nitrogen during the synthesis of N-GQDs. In addition, based on the static quenching effect, the fluorescence quenching of N-GQDs occurred with the increase in CLB concentration. The fluorescence sensor of N-GQDs had good linearity (R^2^ = 0.9879) in the concentration range of CLB (5 × 10^−10^~5 × 10^−7^ M), with a wide linear range and a limit of detection (LOD) of 2.083 × 10^−13^ M. More importantly, the recovery of this probe was good for the detection of CLB in beef and chicken meat. Therefore, the N-GQDs fluorescent probes described in this paper have potential applications for the practical analysis of CLBs as well as other risk substances in food, through the synthetic method of green fluorescent probes.

## Figures and Tables

**Table 1:** Recovery and precision (RSD%) study of CLB in real samples (beef and chicken).

	Added (M)	Found (M)	Recovery (%)	RSD (%, N = 3)
Beef	0	0	0	0
	2.5 × 10^−6^	2.63 × 10^−6^	105.2	10.32
	1.0 × 10^−6^	1.01 × 10^−6^	101.0	9.98
	2.5 × 10^−7^	2.46 × 10^−6^	98.4	8.26
Chicken	0	0	0	0
	2.5 × 10^−6^	2.72 × 10^−6^	108.8	6.90
	1.0 × 10^−6^	9.92 × 10^−7^	99.2	8.36
	2.5 × 10^−7^	2.68 × 10^−7^	107.2	10.25

**Table 2:** Comparison of different reported analytical methods for the detection of clenbuterol.

Analytical Method	Detection Limit	Linear Range	Ref.
Liquidchromatography–mass	0.04 g·L^–1^	0.5–5.0 g·L^–1^	[36]
spectrometry (LC–MS)			
SERS	17.0 fg·mL^−1^	/	[10]
HPLC	12.0 ng∙g^−1^	/	[37]
	0.6 ng∙g^−1^	5000–80,000 ng∙g^−1^	[38]
ELISA	0.2 ppb	/	[7]
Gas chromatography–mass			
spectroscopy (GC–MS)	2.40 lg·kg^−1^	/	[8]
Spectrophotometer	0.93 nM	0–600 nM	[39]
Fluorescence	10^−10^ g·mL^−1^	10^−4^–10^−10^ g·mL^−1^	[40]
Fluorescence	3.0 nM	8–200 nM	[41]
Fluorescence	2.083 × 10^−13^ M	5 × 10^−10^–5 × 10^−7^ M	This work

## Data Availability

The data presented in this study are available on request from the corresponding author.
